# The role of long non-coding RNAs and circular RNAs in cervical cancer: modulating miRNA function

**DOI:** 10.3389/fcell.2024.1308730

**Published:** 2024-02-16

**Authors:** Sama Heidari-Ezzati, Parisa Moeinian, Bahar Ahmadian-Nejad, Faezeh Maghbbouli, Sheida Abbasi, Mahlagha Zahedi, Hamed Afkhami, Alireza Shadab, Nayereh Sajedi

**Affiliations:** ^1^ School of Nursing and Midwifery, Bonab University of Medical Sciences, Bonab, Iran; ^2^ Department of Medical Genetics and Molecular Biology, School of Medicine, Iran University of Medical Sciences, Tehran, Iran; ^3^ School of Nursing and Midwifery, Tehran Medical Branch, Islamic Azad University, Tehran, Iran; ^4^ Tabriz Islamic Azad University of Medical Sciences, Tabriz, Iran; ^5^ Department of obstetrics and gynecology, School of Medicine, Tehran University of Medical Sciences, Tehran, Iran; ^6^ Department of Pathology, Faculty of Medicine, Shahid Sadoughi University of Medical Sciences, Yazd, Iran; ^7^ Nervous System Stem Cells Research Center, Semnan University of Medical Sciences, Semnan, Iran; ^8^ Department of Medical Microbiology, Faculty of Medicine, Shahed University, Tehran, Iran; ^9^ Department of Immunology, School of Medicine, Semnan University of Medical Sciences, Semnan, Iran; ^10^ Iran University of Medical Sciences, Deputy of Health, Tehran, Iran; ^11^ Department of Anatomy, Faculty of Medicine, Qom Medical Sciences, Islamic Azad University, Qom, Iran

**Keywords:** cervical cancer, long non-coding RNAs, circular RNAs, miRNA, microRNAs

## Abstract

Cervical cancer (CC) is a primary global health concern, ranking as the fourth leading cause of cancer-related death in women. Despite advancements in prognosis, long-term outcomes remained poor. Beyond HPV, cofactors like dietary deficiencies, immunosuppression, hormonal contraceptives, co-infections, and genetic variations are involved in CC progression. The pathogenesis of various diseases, including cancer, has brought to light the critical regulatory roles of microRNAs (miRNAs), long non-coding RNAs (lncRNAs), and circular RNAs (circRNAs). The aberrant expression of these miRNAs, lncRNAs, and circRNAs plays a pivotal role in the initiation and progression of CC. This review provides a comprehensive summary of the recent literature regarding the involvement of lncRNAs and circRNAs in modulating miRNA functions in cervical neoplasia and metastasis. Studies have shown that lncRNAs and circRNAs hold great potential as therapeutic agents and innovative biomarkers in CC. However, more clinical research is needed to advance our understanding of the therapeutic benefits of circRNAs and lncRNAs in CC.

## Graphical Abstract

**Figure:**
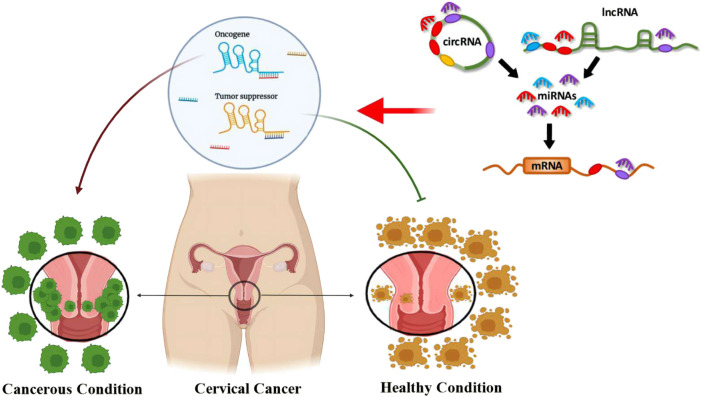
The role of lncRNAs and circRNAs in cervical cancer.

## Introduction

Uterine cervix carcinoma ranks among the most frequently diagnosed gynecological malignancies and is recognized as the fourth most fatal cancer in women on a global scale (He et al., 2020). In 2020, the approximate incidence, prevalence, and fatality rate accounted for 604,127, 1.495.211, and 341,831, respectively (Bray et al., 2018; Singh et al., 2023). Cervical cancer (CC) predominantly manifests in two primary histological forms: squamous cell carcinomas (SCC) and adenocarcinoma (ADC). Adenosquamous carcinoma (ADSC) is the less common type of CC (Nicolás-Párraga et al., 2017; Small et al., 2017). Notably, there has been an increased upsurge in ADC incidence, which is more prevalent among young adult women (Fujiwara et al., 2014). The primary risk factor contributing to CC is the presence of oncogenic human papillomavirus (HPV) (Savira et al., 2022). Previous studies have revealed that HPV 16 and 18 types account for approximately 70% of HPV infections (Aalijahan and Ghorbian, 2019). HPV18 is particularly common in individuals with ADC, while HPV16 is predominant in SCC cases (He et al., 2020). Despite advancements in CC prognosis, long-term outcomes remain poor due to resistance and recurrence (He et al., 2020). Addressing this issue necessitates further research to identify new biomarkers for precise CC progression monitoring and therapeutic targets to enhance survival rates.

The progression from HPV infection to cancer necessitates the involvement of other cofactors, including specific dietary deficiencies, immunosuppression, prolonged use of hormonal contraceptives, co-infection with *Chlamydia trachomatis* (CT), co-infection with herpes simplex virus type 2 (HSV-2) and HIV, high parity, and tobacco smoking, among other potential cofactors (Aalijahan and Ghorbian, 2019). Furthermore, individual genetic variations and distinct epigenetic modifications are pivotal in carcinogenesis. DNA methylation, histone modifications, and non-coding RNAs (ncRNAs) influence gene expression without altering the DNA sequence. The ncRNAs are prevalent throughout the entire genome and can be classified into two subclasses: small ncRNAs with 20–200 nucleotides and long ncRNAs (lncRNAs) with over 200 nucleotides (Hosseini et al., 2017).

### Biogenesis of lncRNAs, circRNAs, and microRNAs

The biogenesis of lncRNAs can be divided into various categories, including exonic, intronic, intergenic, and overlapping (Hosseini et al., 2017). The lncRNAs, through both cis- and trans-regulation, can alter the phenotypes of cancer cells by targeting their final genes (Guttman and Rinn, 2012). Given their capacity to induce dysregulation in various human diseases and foster cancer development, lncRNAs hold significant potential as active biomarkers and, specifically, as therapeutic targets in cancer (He et al., 2020).

Small non-coding RNAs, particularly microRNAs (miRNAs), have been identified as key players in various cellular processes such as cell cycle regulation, inflammation, apoptosis, migration, and differentiation. Additionally, they influence messenger RNA (mRNA) translation and stability (Di Leva et al., 2014). Studies have substantiated that miRNAs can function as oncogenes or tumor suppressors in various cancer types (Romano et al., 2017; Rupaimoole and Slack, 2017). Understanding the regulatory mechanisms governing miRNA expression is closely intertwined with cancer diagnosis, prognosis, treatment, and underlying pathogenesis (Shen et al., 2020).

Circular RNAs (circRNAs) are a large family of non-coding RNAs in the transcript of eukaryotes. They appear as covalently closed loops formed by successive exons in the nucleus. CircRNAs have polar 5′ and 3′ ends and polyadenylated tails. There are many types of circRNAs in different cell lines and species, involving diverse ranges of biological processes between cells (Chen and Yang, 2015). After transferring to the cytoplasm, circRNAs perform various biological actions by binding to microRNAs and proteins (Ren et al., 2019). They play a vital role in transcriptional regulation and are potential biomarkers (Liang et al., 2021). Additionally, circRNAs are secreted into body fluids both in free form and encapsulation in multi-vesicular endosomes and exosomes along with lipids, proteins, and nucleic acids, controlling various biological functions in different areas of the body (Wang et al., 2019a), including fetal growth and brain synaptic plasticity (Liang et al., 2021). Moreover, abnormal expression of circRNAs is detected in the occurrence of diseases such as human malignancies, Alzheimer’s, and Parkinson’s disease (Nisar et al., 2021).

### Biological functions of long non-coding RNAs

Long non-coding RNAs exhibit a broad spectrum of biological and regulatory functions, including proliferation, differentiation, development, and DNA damage repair (Wang and Chang, 2011). They interfere with transcription, translation, and post-transcriptional and post-translation processes, enabling a direct interaction with DNA, RNA, and proteins (He et al., 2020). Cis-acting lncRNAs are closely associated with specific transcription sites, affecting neighboring genes’ chromatin structure or expression state on the same allele. In contrast, trans-acting lncRNAs act from a distance, triggering the expression of genes, proteins, or RNA molecules within the cell (Guttman and Rinn, 2012; Kopp and Mendell, 2018).

LncRNAs are involved in epigenetic gene regulation through three main mechanisms. First, they influence the transcription of target genes by regulating histone modifications and the chromatin status. Second, lncRNAs actively recruit repressors or cell transcription factors to the promoters of their target genes. Third, they tightly bind to proteins involved in transcription, preventing them from attaching to their specific DNA targets (Long et al., 2017).

**FIGURE 1:**
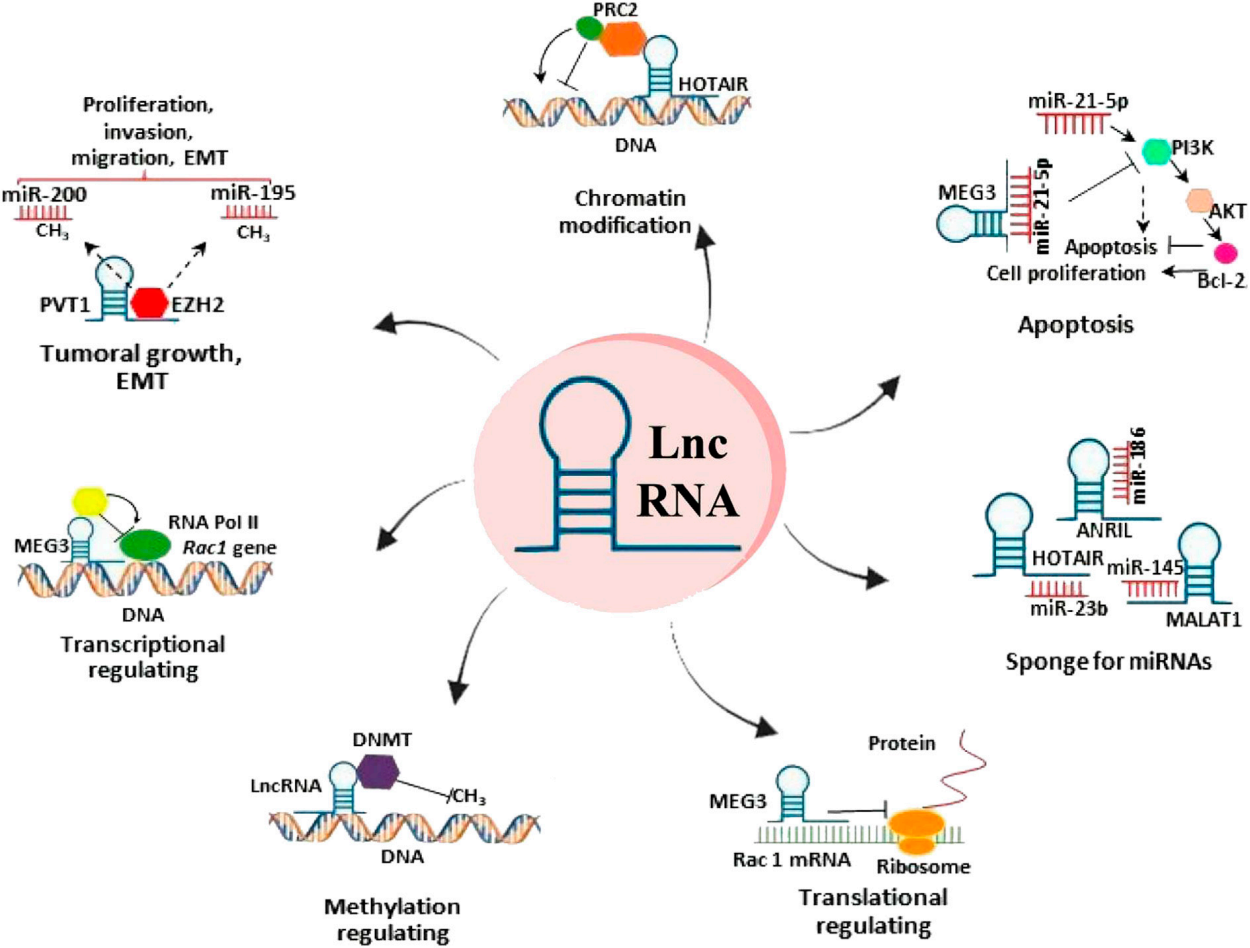
Functions of long non-coding RNAs (lncRNAs) in cervical cancer.

In addition, lncRNAs act as competing endogenous RNAs (ceRNAs) by binding to miRNAs, thereby preventing miRNA-mediated gene suppression (Gutschner and Diederichs, 2012). Some lncRNAs serve as templates or precursors for various small RNAs (Cheetham et al., 2013) and influence alternative mRNA splicing (Romero-Barrios et al., 2018). At the post-translational level, lncRNAs can bind to target proteins, affecting their localization and transportation. Previous studies have reported that lncRNAs function as scaffolds, facilitating protein–protein interactions, and the formation of protein complexes (Yoon et al., 2013) (Figure 1).

### Relevant lncRNAs in cervical cancer

Long non-coding RNAs interact with proteins, mRNAs, and miRNAs and play a critical role in cancer progression. Several lncRNAs, including HOTAIR, CCAT2, H19, MALT1, SPRY4-IT1, PVT1, GAS5, MEG3, and C5orf66-AS1, have been identified as critical players in the development, invasion, metastasis, and radio resistance of CC (see Table 1) (Larrieta-Carrasco et al., 2018; Tornesello et al., 2020).

**TABLE 1:** Related lncRNAs and functions in cervical cancer.

LncRNA	Histologic type	Function	Sponged miRNAs	Modulated pathways in cervical cancer	References
HOTAIR	SCC/ADC	Oncogenic	miR-22, miR-23b, and miR-143-3p	BCL2, PRC2, LSD1, VEGF, mmP-9, mTOR, Notch, Wnt, STAT3, wnt/β-catenin, PI3K/AKT, and HPV E7 oncoprotein	Gutschner and Diederichs (2012), Guttman and Rinn (2012), Jiang et al. (2014), Gao et al. (2017), Hosseini et al. (2017), Hsiao et al. (2017), Gao et al. (2019), He et al. (2020), Ji et al. (2020), and Ho et al. (2021)
CCAT2	SCC/ADC	Oncogenic	miR-17-5p and miR-20a	MYC and wnt in colon cancer	Kallen et al. (2013) and Kim et al. (2015)
CCAT1	SCC/ADC	Oncogenic	_	MMP14 and Wnt/β-catenin pathway by c-myc	Kopp and Mendell (2018) and Knupp et al. (2021)
H19	–	Oncogenic	miR-138-5p	IGF2, HPV E6 oncoprotein, and upregulate SIRT1	Lee et al. (2016), Kopp and Mendell (2018), Lecerf et al. (2019), and Li et al. (2020a)
MALAT1	–	Oncogenic	miR-124, miR-145, and miR-206	RBG2, E-cadherin, β-catenin, vimentin, ZO-1, caspase-3, caspase-8, Bax, Bcl-2, and BclxL	Li et al. (2017), Li et al. (2018), Li et al. (2019), and Li et al. (2020b)
SPRY4-IT1	SCC/ADC	Oncogenic	miR-101-3p	ZEB1, EMT, E-cadherin, and vimentin	Liu et al. (2018b), Liang et al. (2021), and Liu and Li (2023)
PVT1	–	Oncogenic	miR-200, miR-424, and miR-195	EZH2, Myc, Nop2, p15, p16, H3K27me3, NF-kB, and repression of miR-16 via the NF-κB pathway	Lu et al. (2016), Liu et al. (2017), Long et al. (2017), and Liu et al. (2018a)
GAS5	SCC/ADC	Tumor-suppressive	miR-106b, miR-196a, and miR-205	IER3, FOXO1, and PTEN expression	Nicolás-Párraga et al. (2017), Ma et al. (2018), Meng et al. (2021), and Nisar et al. (2021)
MEG3	_	Tumor-suppressive	miR-21-5p	P-STAT3	Paci et al. (2014), Peng and Fan (2016), Romano et al. (2017), Ou et al. (2018), Qi et al. (2019), and Ren et al. (2019)
C5orf66-AS1	_	Oncogenic	miR-637	RING1	Rüegg (1985) and Romero-Barrios et al. (2018)
LINC00675	_	Oncogenic	_	Wnt/β-catenin, Bax, and GSK-3β Bcl-2	Zhang et al. (2021a)
FAM83H-AS1	_	Oncogenic	_	HPV E6 and E6-p300	Barr et al. (2019)
RSU1P2	_	Oncogenic	let-7a	IGF1R and N-myc	Liu et al. (2017)
NOC2L-4.1	_	Oncogenic	miR-630	YAP1	Wang et al. (2019b)
PAX8 AS1	_	Tumor-suppressive	_	PAX8 and NOTCH1 (pancreatic carcinoma)	Qi et al. (2019)
EBIC	_	Oncogenic	_	EZH2, Wnt/β-catenin, and E-cadherin	Sun et al. (2014) and Xu et al. (2018)
CCHE1	SCC	Oncogenic	_	PCNA and ERK/MAPK	Yang et al. (2015) and Peng and Fan (2016)
LET	_	Tumor-suppressive	_	LIN28	Tornesello et al. (2020)

Squamous cell carcinoma, SCC; adenocarcinoma, ADC.

### HOTAIR lncRNA

HOTAIR, “2.2 kb HOX transcript antisense intergenic RNA,” is one of the extensively studied oncogenic lncRNAs. It is encoded by the antisense strand of the *HOXC* gene and is located on chromosome 12 at position q13.13 (Rüegg, 1985). Like other lncRNAs, HOTAIR recruits chromatin-modifying proteins and influences the epigenome of cancer (Ren et al., 2019). In CC, the levels of HOTAIR are tightly controlled by the HPV E7 protein (Sharma et al., 2015). Furthermore, HOTAIR functions as a sponge for specific miRNAs, resulting in the dysregulation of their respective target genes. For instance, HOTAIR has been reported to alter the miR-143-3p/BCL2 axis, promoting CC cell growth (Liu et al., 2018a). It also modulates the miR-23b/MAPK1 axis, which is involved in cell proliferation and metastasis (Li et al., 2018). Additionally, HOTAIR overexpression has regulatory effects on various metabolic functions, such as the activation of the Notch-Wnt signaling pathway in SiHa cells (Lee et al., 2016) and the mTOR pathway in different CC cell lines, including C33A, HeLa, and CaSki (Zhang et al., 2015). The expression of HOTAIR leads to the upregulation of matrix metalloproteinase-9 (MMP-9), vascular endothelial growth factor (VEGF), and genes associated with the epithelial–mesenchymal transition (EMT), thereby promoting tumor aggressiveness in CC (Kim et al., 2015). It is worth noting that the HOTAIR enhancer contains a single nucleotide polymorphism, rs920778T, which is associated with HOTAIR overexpression and increased susceptibility to cancer (Xiao et al., 2011). In cases of HPV-positive CC, the rs920778 C HOTAIR allele is more prevalent, but its expression level may be reduced due to the binding of miR22 to the rs920778C sequence, leading to the suppression of the HOTAIR expression (Sh et al., 2016).

### CCAT2 lncRNA

Substantial upregulation of the long non-coding RNA, known as colon cancer-related transcript 2 (CCAT2), has been observed in CC cells, including HeLa, SiHa, and CaSki, as well as in CC tissues (Lee et al., 2016).

### CCAT1 lncRNA

The lncRNA CCAT1 is located within the c-Myc gene region, belonging to the Myc proto-oncogene family, which is known for its critical role in tumorigenesis. CCAT1, acting as a sponge for miR-181a-5p, promotes the proliferation and invasion of CC cells. This function is accomplished through the upregulation of heparin-binding EGF-like growth factor (HB-EGF) and matrix metalloproteinase-14 (MMP14) (Shen et al., 2019). Furthermore, upon binding to c-Myc, CCAT1 activates the Wnt/β-catenin signaling pathway, leading to increased cell progression (Zhang and Gao, 2017).

### H19 lncRNA

The lncRNA H19 is encoded by the H19 gene and is situated in chromosome 11 at 11p15.5. It is expressed exclusively from the maternally inherited chromosome and is recognized as the first riboregulator lncRNA. H19 is expressed in various tissues, including fetal tissues, adult muscles, and cancers (Kallen et al., 2013). The HPV16 E6 oncoprotein modulates lncRNA H19, which acts as a molecular sponge for miR-138-5p in epithelial cells (Ou et al., 2018; Barr et al., 2019). However, H19 promotes cell proliferation and inhibits apoptosis by silencing miR-143-3p and upregulating sirtuin-1 (SIRT1) (Ou et al., 2018).

### Metastasis-associated lung adenocarcinoma transcript 1 lncRNA

The metastasis-associated lung adenocarcinoma transcript 1 (MALAT1) consists of 8,000 nucleotides and is located in chromosome 11q13.1. It was first recognized in non-small cell lung cancer (Sun and Ma, 2019). Overexpression of MALAT1 has been observed in CC cell lines and cancer tissues infected with high-risk HPV (Jiang et al., 2014). MALAT1 is a sponge for several miRNAs, including miR-124, miR-145, and miR-20 (Lu et al., 2016). Consequently, downregulation of MALAT1 in both CC tissues and CC cell lines leads to a significant reduction in invasion and metastasis, achieved through the modulation of the MALAT1-miR-124-RBG2 axis and the inhibition of the epithelial–mesenchymal transition (Lee et al., 2016). Additionally, MALAT1 plays a role in the mechanisms of radio resistance by acting as a sponge for miR-145 during CC radiotherapy (Lu et al., 2016).

### SPRY4-IT1 lncRNA

Intron two of the *SPRY4* gene generates SPRY4 intronic transcript 1 (SPRY4-IT1), a molecule that assumes the role of either a tumor suppressor or an oncogenic factor in various cancer types (Xie et al., 2015). However, in CC, SPRY4-IT1 expression levels surpass those of normal tissues and are closely associated with advanced clinical stages, ultimately diminishing the survival rates of CC patients (Cao et al., 2016). Recent research has unveiled the pivotal role of SPRY4-IT1 silencing in CC cell lines, particularly in inhibiting migration and invasion via the SPYR4-IT1/miR-101-3p/ZEB1 axis. This function is directly linked to the active suppression of epithelial–mesenchymal transition (EMT) alterations, leading to increased E-cadherin levels and reduced expression levels of both N-cadherin and vimentin (Fan et al., 2019).

### PVT1 lncRNA

Plasmacytoma variant translocation 1 (PVT1) is an exceptionally conserved long non-coding RNA (lncRNA) located downstream of the MYC gene. It is frequently co-amplified with MYC in various types of cancer (Li et al., 2019). PVT1 downregulates the expression levels of miR-195 and miR-200b in CC through direct binding or the enhancement of histone H3K27me3 in the promoter regions of miR-195 and miR-200b (Zhang et al., 2016; Shen et al., 2017). MiR-195 is associated with chemoresistance and the epithelial–mesenchymal transition, while miR-200b plays a role in cellular proliferation, invasion, and migration of CC cells. A positive correlation between serum PVT1 levels and the expression of PVT1 in cancer tissues has been reported, suggesting PVT1 as a potential diagnostic biomarker for CC (Yang et al., 2016).

### GAS5 lncRNA

Growth arrest-specific transcript 5 (GAS5) exhibits tumor suppressor activity in various cancer types (Schneider et al., 1988; Pickard and Williams, 2016). Therefore, in CC, lower GAS5 expression levels are linked to tumor progression and poorer clinical outcomes for individuals with CC (Cao et al., 2014). The inhibition of GAS5 in CC cells has been observed to enhance proliferation, migration, and invasion, further underscoring its potential as a tumor suppressor in the development of CC (Cao et al., 2014). Notably, GAS5 functions as a sponge for miR-106b, resulting in the increased expression of IER3 (immediate early response 3) and enhanced radio sensitivity of CC cells (Gao et al., 2019).

### Maternally expressed gene 3 lncRNA

Maternally expressed gene 3 (MEG3), a long non-coding RNA derived from the DLK1-MEG3 locus at the chromosomal location 14q32.3, is well-established as a cancer-suppressive factor (Zink et al., 2018), which was initially recognized as the ortholog of gene trap locus 2 (Gtl2) in mice. MEG3 overexpression demonstrates preventive effects on cell proliferation and enhances apoptosis in cancer cells, both in a p53-dependent and independent manner (Paci et al., 2014). A notable reduction in the MEG3 expression level was observed in CC tissues and cell lines. Enhancing MEG3 in CC cell lines prevented proliferation and induced cell cycle arrest and apoptosis. It was achieved by direct binding to P-STAT3, leading to subsequent ubiquitination and degradation of P-STAT3 (Zhang and Gao, 2019). Additionally, MEG3 exerts its cancer-suppressive effect by downregulating the levels of miR-21-5p in CC cell lines (Avila et al., 2023). Knockdown of MEG3 in HeLa and CaSki cells resulted in a significant upregulation of miR21-5p expression (Yadav et al., 2023).

### C5orf66-AS1 lncRNA

C5orf66-AS1 lncRNA primarily resides in the cytoplasm, suggesting its modulation of CC cell functions through the competing endogenous RNA (ceRNA) mechanism (Begliarzade et al., 2023). Previous research has identified interactions between C5orf66-AS1 and various miRNAs, including miR-621, miR-637, miR-663b, miR-3184, miR-3187, miR-4449, miR-4706, and miR-5001, depicting its role as a sponge for these miRNAs. Alterations in the expression levels of C5orf66-AS1 in CC cells (C-4 I and SiHa) were observed to correlate with changes in the miR-637 expression. Specifically, the upregulation of C5orf66-AS1 led to a significant decrease in miR-637 expression levels and *vice versa* (Rui et al., 2018).

### Novel lncRNAs in cervical tumorigenesis

**FIGURE 2:**
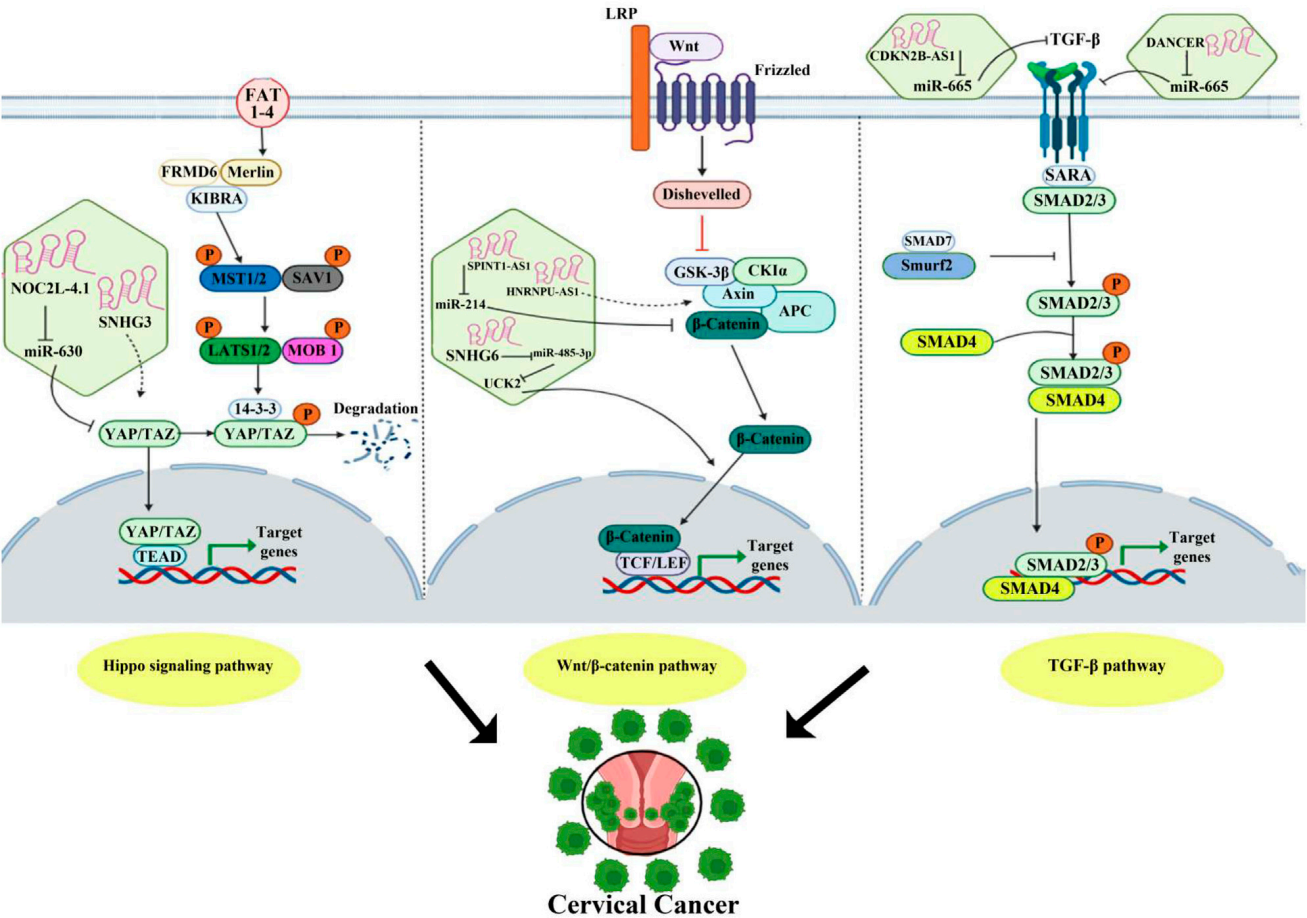
LncRNA effects in metabolic pathways in cervical cancer.

In addition to the mentioned lncRNAs, several novel sequences of lncRNAs, such as LINC00675, FAM83H-AS1, RSU1P2, NOC2L-4.1, EBIC, PAX8 AS1, CCHE1, and LET, have been identified in cervical tumorigenesis (refer to Table 1) (Figure 2).

### Biological functions of circular RNAs

**FIGURE 3:**
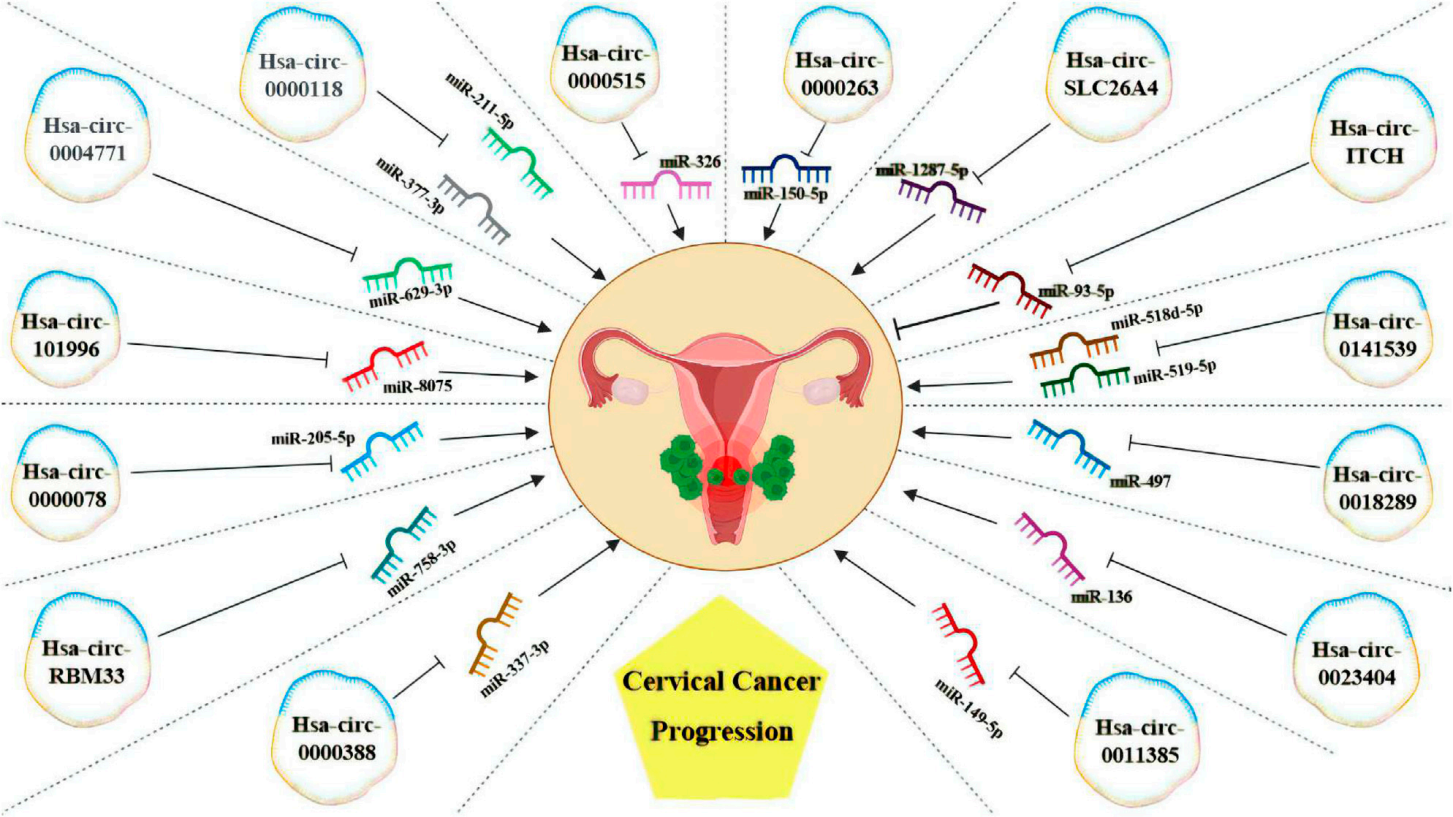
Functions of circular RNAs (circRNAs) in cervical cancer.

Circular RNAs (circRNAs) represent a recently discovered class of widespread endogenous non-coding RNA molecules characterized by their covalent circular structures, which serve as transcription factors, miRNA sponges, and RNA-binding proteins (RBPs) (Hsiao et al., 2017). CircRNAs have been observed to exhibit abnormal expression patterns in cancer cells and play diverse roles in the development and progression of cancer. Due to their unique stable loop-like structure, circRNAs have shown promise as potential biomarkers and therapeutic targets for various types of cancer (Zhu et al., 2021). CircRNAs have various biological functions such as regulation of gene expression (Hansen et al., 2013). They act as microRNA sponges, sequestering and inhibiting the activity of microRNAs, thus modulating the expression of target genes (Hansen et al., 2013). CircRNAs can interact with proteins (Du et al., 2016) and regulate alternative splicing of mRNA by interacting with splicing factors, thereby regulating the diversity of the transcriptome (Ashwal-Fluss et al., 2014). They also play roles in cellular proliferation, apoptosis, and cellular senescence (Abdelmohs et al., 2015; Li et al., 2015). CircRNAs are also associated with various aspects of cancer, including tumorigenesis, metastasis, and resistance to therapy (Guarnerio et al., 2016). Additionally, they have been implicated in neurological disorders, synaptic function, neuronal development, and neurodegenerative diseases (Rybak-Wolf et al., 2015) (Figure 3).

### Regulation of circRNA biogenesis

CircRNA biogenesis is intricately controlled by repetitive intronic elements, particularly sequences derived from transposons (Wilusz, 2015). The production of circRNA involves alternative splicing of one or several exons of a gene in the presence of an RNA-mediated silencing complex (Liang et al., 2021). Splice sites bring exons closer together and facilitate the formation of closed loops (Wilusz, 2015). Backsplicing, mediated by inversion repeats flanking cyclic exons, further aids circRNA production. When producing circRNAs, different versions of the closed loop are created depending on repeated base pairs. Therefore, the specific arrangement of intronic repeats regulates the potential function of protein-coding genes (Wilusz, 2015).

### Protein factors in circRNA biogenesis

Numerous protein factors play pivotal roles in circRNA biogenesis. NF90/NF110 is a crucial regulator that assembles exons to form circRNAs in the nucleus and interacts with mature circRNAs in the cytoplasm (Li et al., 2017). NOVA2, an enriched RNA-binding protein, plays a crucial role in the circRNA expression by binding to specific sites inside the introns flanking circRNA exons (Knupp et al., 2021). Heterogeneous nuclear ribonucleoprotein M (HNRNPM) is another factor involved in controlling circRNA biogenesis by preventing misplaced exon splicing and backsplicing after binding to homeostatic gene transcripts (Ho et al., 2021). The RNA-binding protein SFPQ regulates circRNA biogenesis by overseeing long-intron splicing, ensuring precise splicing, and preserving the intron integrity (Stagsted et al., 2021).

### Circular RNA and modulation microRNAs in cervical cancer

circRNAs have emerged as a novel therapeutic target in CC (Chaichian et al., 2020). A recent study highlighted the role of circRNA-VPRBP in blocking galectin-1-mediated CC metastasis by binding to RACK1 O-GlcNAcylation (Zhang et al., 2023). Aberrant expression of circRNAs has been linked to the initiation of pathological processes in cervical tissue, influencing cell proliferation, migration, and apoptosis. CircRNAs are considered a potential clinical biomarker for early detection of CC, offering a prognosis for early treatment (Chen et al., 2021). Understanding the circRNA–miRNA–mRNA network’s role in CC pathogenesis opens avenues for designing chemotherapy drugs targeting this network, providing new possibilities for CC treatment (Yi et al., 2019).

Several circRNAs act as competing endogenous RNAs or microRNA sponges, regulating mRNA expression (Arnaiz et al., 2019). For instance, circRNA hsa_circ_0000515 acts as a sponge for miR-326, mediating CC progression. Silencing hsa_circ_0000515 reduces proliferation and invasion while promoting apoptosis and autophagy in CC cells. Another study revealed that hsa_circ_0000263 knockdown suppresses cell migration and proliferation by controlling the target gene of miR-150-5p (Cai et al., 2019). Hsa-circSLC26A4 accelerates CC progression via the QKI/circSLC26A4/miR-1287-5p/HOXA7 axis (Ji et al., 2020). Overexpression of circRNA hsa-Circ-ITCH inhibits malignant behaviors in CC by regulating microRNA-93-5p and forkhead box K2 (FOXK2) expression (Li et al., 2020a).

Hsa_circ_0141539, acting as a sponge for miR-518d-5p/519-5p, leads to increased CBX8 expression, promoting malignant transformation and proliferation of cervical cells (Liu et al., 2018b). Additionally, this circRNA has been shown to sponge miR-506, resulting in the upregulation of Snail-2 expression, a direct target of miR-506 (Ma et al., 2018). Gao et al. demonstrated that Hsa_circ_0018289 binds to miR-497, enhancing invasion, cell proliferation, and migration of CC cells (Gao et al., 2017). Hsa_circ_0023404 acts as a miR-136 sequester, promoting the upregulation of TFCP2 expression, activating the YAP signaling pathway, and contributing to CC progression (Zhang et al., 2018). Hsa_circ_0011385 increased expression in CC influences SOX4 expression by targeting miR-149-5p, affecting the malignant biological behaviors of CC cells (Xu et al., 2021). Hsa_Circ_0000388 accelerates CC progression by modulating miR-337-3p and TCF12 expression (Meng et al., 2021). Circ_0043280 regulates CC through the miR-203a-3p/PAQR3 axis, competitively binding miR-203a-3p and restoring PAQR3 expression, thereby preventing tumor growth and metastasis (Zhang et al., 2021b).

Circular RNA (hsa_circ_0001772) is implicated in cancer tumorigenesis, and its knockdown reduces tumor growth, migration, invasion, and glycolysis and promotes CC cell apoptosis (Ding et al., 2021). Hsa_circ_0001772 functions as a sponge for miR-758-3p, modulating PUM2 expression, which reverses the suppressive influence of upregulated miR-758-3p on CC cell malignant behaviors. Hsa_circ_0001772 fosters CC advancement by absorbing miR-758-3p and enhancing PUM2 expression.

In another study, circRNA-0000078 was found to suppress CC cell survival and proliferation by regulating the miRNA-205-5p/EREG pathway, preventing tumorigenesis development in this cancer type (Liu and Li, 2023). Hsa_circRNA_101996 promotes the proliferation and invasion of cancer by increasing TPX2 factor expression, inhibiting miR-8075, and is directly correlated with the tumor size and lymph node metastasis (Stagsted et al., 2021). CircRNA-SLC26A4 plays a crucial role in promoting CC proliferation and invasion by targeting the QKI/circSLC26A4/miR-1287-5p/HOXA7 axis. CircRNA_0018289 contributes to cervix tumorigenesis by targeting miRNA-497 (Gao et al., 2017). CircRNA-NRIP1 (Hsa-circ-0004771) enhances CC oncogenic effects and lymph node invasion by impacting miRNA-629-3p and subsequently on the PTP4A1/ERK1/2 signaling pathway (Li et al., 2020b). CircRNA-00284 arrests the cell cycle at the G0/G1 phase, increasing CC invasion through miRNA-506 sponging and Snail-2 upregulation (Ma et al., 2018). Hsa-circRNA-0000118 is implicated in cervical cancer malignancy by targeting miR-211-5p and miR-377-3p, preserving AKT2 levels (Wu et al., 2023) (Table 2).

**TABLE 2:** Related circRNAs and functions in cervical cancer.

CircRNA	Function	Sponged miRNA	Modulated pathways in cervical cancer	References
Hsa-circ-0000515	Oncogenic	miR-326	ELK1, Caspase3, Caspase9, MMP-9, TIMP-1, Beclin1, P62, LC3-I, and LC3-II	Tang et al. (2019)
Hsa-circ-0000263	Oncogenic	miR‐150‐5p	MDM4 and p53 expression	Cai et al. (2019)
Hsa-circ-SLC26A4	Oncogenic	miR-1287-5p	HOXA7	Ji et al. (2020)
Hsa-circ-ITCH	Tumor-suppressive	microRNA-93-5p	Forkhead box K2 (FOXK2)	Li et al. (2020a)
Hsa-circ-0141539	Oncogenic	miR-518d-5p/519-5p	CBX8 protein	Liu et al. (2018b)
Hsa-circ-0018289	Oncogenic	miR-497	-	Gao et al. (2017)
Hsa-circ-0023404	Oncogenic	miR-136	TFCP2 and YAP expression	Zhang et al. (2018)
Hsa-circ-0011385	Oncogenic	miR-149-5p	SOX4 expression	Xu et al. (2021)
Hsa-circ-0000388	Oncogenic	miR-337-3p	TCF12	Meng et al. (2021)
Hsa-circ-RBM33	Oncogenic	miR-758-3p	Pumilio RNA-binding family member 2 (PUM2)	Ding et al. (2021)
Hsa-circRNA-0000078	Oncogenic	miR-205-5p	-	Liu and Li (2023)
Hsa-circRNA_101996	Oncogenic	miR-8075	TPX2 expression	Song et al. (2019)
circRNA-NRIP1 (Hsa-circ-0004771)	Oncogenic	miR-629-3p	PTP4A1/ERK1/2 pathway	Li et al. (2020b)
Hsa-circRNA-0000118	Oncogenic	miR-211-5p/miR-377-3p	AKT2 expression	Wu et al. (2023)

## Conclusion and future directions

Due to various limitations and shortcomings, such as challenges in early detection, a lack of targeted therapies, limited HPV vaccination coverage, treatment side effects, and resistance to therapy, CC stands as one of the leading causes of death among women worldwide. LncRNAs and circRNAs serve pivotal roles in modulating miRNA activity, thereby influencing the initiation and progression of cervical carcinogenesis. These non-coding RNAs engage in intricate regulatory networks, directly interacting with miRNAs to impact downstream target mRNA expression. Examples of such interactions include lncRNA HOTAIR, which acts as a sponge for miR-23b-3p, relieving the suppression of its target gene ZEB1. This leads to enhanced EMT and increased metastatic potential in CC cells. Similarly, circRNA CDR1as sequesters miR-7, leading to increased expression of its target gene EGFR, promoting cell proliferation and migration in CC. The dysregulation of these non-coding RNA-mediated interactions contributes significantly to key aspects of cervical carcinogenesis, including uncontrolled proliferation, evasion of apoptosis, and increased metastatic potential. Furthermore, the intricate interplay between lncRNAs, circRNAs, and miRNAs contributes to the establishment of a conducive tumorigenic microenvironment. Numerous studies have explored the diagnostic and therapeutic potential of miRNAs in various cancers. This review focuses on summarizing the role of lncRNAs and circRNAs in the context of CC. Understanding these regulatory mechanisms at the molecular level provides valuable insights into the complexity of CC pathogenesis. Targeting the dysregulated interactions between lncRNAs, circRNAs, and miRNAs holds promise for developing innovative diagnostic and therapeutic strategies against cervical carcinogenesis. Thus, elucidating these molecular networks enhances our comprehension of CC biology and informs potential avenues for precision medicine in its management. Additionally, the review emphasizes the potential of lncRNAs as biomarkers and therapeutic targets in CC. It provides a detailed examination of specific lncRNAs implicated in CC development, such as HOTAIR, CCAT2, H19, MALAT1, SPRY4-IT1, PVT1, GAS5, MEG3, and C5orf66-AS1. We present a comprehensive list of circRNAs and their functions in CC, detailing how they act as miRNA sponges, influencing mRNA expression, and contributing to the pathogenesis of CC.

This article is a valuable resource for researchers, highlighting the potential of lncRNAs and circRNAs as promising targets for diagnostic and therapeutic interventions in CC. Efforts to identify early detection biomarkers in CC focus on specific lncRNA–miRNA–target mRNA networks associated with initial stages of the disease. The dysregulation of a lncRNA, which modulates miRNA activity and influences the expression of oncogenic mRNAs, holds potential as an early diagnostic biomarker, enabling timely intervention and improving prognosis. Investigating diverse interactions within lncRNA–miRNA–target mRNA networks in CC subtypes can lead to the development of molecular signatures for precise subtyping. These specific networks may indicate distinct subtypes, aiding in the formulation of tailored treatment strategies and enhancing therapeutic outcomes. Additionally, exploring liquid biopsy approaches to detect lncRNAs, miRNAs, and target mRNAs in bodily fluids offers non-invasive diagnostic possibilities. Circulating nucleic acids associated with regulatory networks can serve as liquid biopsy markers, providing a convenient method for monitoring disease progression and treatment response. Integrating diverse lncRNA–miRNA–target mRNA networks with established molecular markers contributes to comprehensive molecular profiling, enhancing CC diagnosis accuracy by considering multiple layers of molecular information. This understanding guides clinicians in developing personalized treatment plans. Furthermore, identifying specific lncRNA–miRNA–target mRNA networks linked to treatment response or resistance serves as therapeutic monitoring markers. Monitoring dynamic changes in these networks provides real-time insights, allowing for timely adjustments in treatment strategies. In conclusion, recognizing the potential of lncRNAs and circRNAs as diagnostic markers in CC prompts specific exploration of molecular networks for practical clinical applications. Spanning from early detection to subtype-specific diagnostics and non-invasive monitoring, these applications offer actionable directions for translating molecular discoveries into tangible benefits for CC patients. However, the utilization of these emerging lncRNAs and circRNAs as biomarkers for both the prognosis and diagnosis of CC requires further exploration through extensive clinical research in this field.
